# Phosphatidylcholine Ameliorates Palmitic Acid‐Induced Lipotoxicity by Facilitating Endoplasmic Reticulum and Mitochondria Contacts in Intervertebral Disc Degeneration

**DOI:** 10.1002/jsp2.70062

**Published:** 2025-03-31

**Authors:** Shuangshuang Tu, Yijun Dong, Chuanfu Li, Mingxin Jiang, Liqun Duan, Wenzhi Zhang, Xi Chen

**Affiliations:** ^1^ Department of Orthopedics, The First Affiliated Hospital of USTC, Division of Life Sciences and Medicine University of Science and Technology of China Hefei China; ^2^ College of Pharmacy, Anhui Xinhua University Hefei China; ^3^ Department of Orthopedics The First Affiliated Hospital of USTC, Provincial Hospital Affiliated to Anhui Medical University Hefei China; ^4^ Clinical College of Anhui Medical University Hefei China; ^5^ School of Clinical Medicine Anhui University of Science and Technology Huainan China

**Keywords:** cell senescence, IDD, lipotoxicity, palmitic acid, phosphatidylcholine

## Abstract

**Background:**

Intervertebral disc degeneration (IDD) is a prevalent musculoskeletal disorder with substantial socioeconomic impacts. Despite its high prevalence, the pathogenesis of IDD remains unclear, and effective pharmacological interventions are lacking. This study aimed to investigate metabolic alterations in IDD and explore potential therapeutic targets by analyzing lipotoxicity‐related mechanisms in nucleus pulposus (NP) cells.

**Methods:**

Metabolomics and magnetic resonance spectroscopy were utilized to profile metabolic changes in NP tissues from advanced‐stage IDD. Transcriptomics and metabolomics integration were performed to identify key regulatory pathways. In vitro experiments using human NP cells exposed to palmitic acid were conducted to evaluate endoplasmic reticulum (ER) stress, mitochondrial dysfunction, lipid droplet accumulation, and senescence. Phosphatidylcholine supplementation was tested for its ability to mitigate lipotoxicity, with ER‐mitochondria interactions and mitochondrial oxidation capacity assessed as mechanistic endpoints.

**Results:**

Our findings revealed an abnormal lipotoxic condition in NP cells from advanced‐stage IDD. Furthermore, we identified abnormal accumulation of triglycerides and palmitic acid in NP cells from IDD. The palmitic acid accumulation resulted in endoplasmic reticulum stress, mitochondrial damage, lipid droplet accumulation, and senescence of NP cells. By integrating transcriptomics and metabolomics analyses, we discovered that phosphatidylcholine plays a role in regulating palmitic acid‐induced lipotoxicity. Notably, phosphatidylcholine level was found to be low in the endoplasmic reticulum and mitochondria of advanced‐stage NP cells. Phosphatidylcholine treatment alleviated palmitic acid‐induced lipid droplet accumulation and senescence of NP cells by modulating ER‐mitochondria contacts and mitochondrial oxidation capacity.

**Conclusion:**

Phosphatidylcholine emerges as a potential therapeutic agent to counteract lipotoxic stress by modulating organelle interactions and mitochondrial function. These findings advance our understanding of IDD pathogenesis and provide a novel metabolic target for therapeutic development.

## Graphical abstract

The abnormal lipotoxic condition in nucleus pulposus (NP) cells from advanced‐stage intervertebral disc degeneration (IDD) was identified through untargeted LC/MS metabolomics and magnetic resonance spectroscopy. The palmitic acid accumulation resulted in endoplasmic reticulum stress, mitochondrial damage, lipid droplet accumulation, and senescence of NP cells; Phosphatidylcholine treatment alleviated palmitic acid‐induced lipid droplet accumulation and senescence of NP cells by modulating ER‐mitochondria contacts and mitochondrial oxidation capacity.

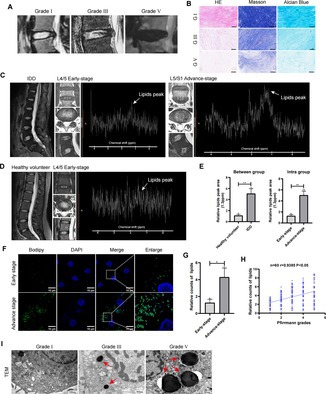

## 1 Introduction

Intervertebral disc degeneration (IDD) is the main cause of chronic low back pain and spine‐related disorders, which imposes significant personal, economic, and social burdens on individuals worldwide [1]. Current management strategies and treatments for IDD, such as analgesics and nonsteroidal anti‐inflammatory drugs, are focused mainly on pain relief but do not arrest disease progression [2]. The existing therapeutic challenge is attributed in part to a restricted comprehension of IDD pathogenesis. Cell senescence in nucleus pulposus (NP) cells, characterized by permanent cell cycle arrest triggered by various stressors, such as oxidative stress, lipotoxicity, and mechanical stress, has been identified as a significant pathophysiological mechanism contributing to IDD [3, 4, 5, 6, 7]. Consequently, there is a pressing need to elucidate the biological events and molecular pathways involved in IDD to advance the development of therapeutic interventions.

The intervertebral disc consists of central NP tissues surrounded by the annulus fibrosus and endplates [7]. Owing to its avascular nature, the nutrient supply and metabolite exchange of the central NP tissues rely primarily on diffusion through the endplates [8]. As the endplates undergo calcification with aging and degeneration, there may be a reduction in diffusion capacity. Decreased diffusion results in a lack of nutrients and an abnormal accumulation of metabolic waste and is considered a major contributing factor for the development of IDD [9]. Prior research has revealed that the excessive accumulation of toxic metabolites, including advanced glycation end products, free fatty acids, and lactate, all directly contribute to the toxic injury and dysfunction of NP cells [10, 11, 12]. Understanding changes in metabolic profiles can provide insight into the biological consequences of metabolite accumulation and may offer new therapeutic approaches for IDD. However, there is limited knowledge about the metabolic profile alterations that occur when NP cells enter senescence.

Metabolites, which are the substrates and products of metabolism, serve as indicators of abnormalities in cell and tissue functions, allowing for a direct assessment of biological event regulation [13]. Therefore, an examination of comprehensive changes in metabolic profiles throughout the progression of IDD may offer valuable insights into the underlying pathological processes, shedding light on the intricate regulatory mechanisms linking pathological insults to NP cell senescence. In our prior investigation, an untargeted LC–MS metabolomics approach revealed aberrant lipotoxic environments in IDD, characterized by the upregulation of lysophosphatidylcholine, palmitic acid (PA), and triglycerides [4]. Lipotoxicity can arise from a variety of environmental and cellular factors, including increased intracellular free fatty acid concentrations, inflammatory processes, elevated levels of reactive oxygen species, and metabolic alterations [14, 15]. Although lipotoxicity is implicated in numerous metabolic and age‐related disorders, its functional role in human IDD remains unexplored, and there is little information concerning the presence of triglycerides and lipid droplets in human NP cells.

Multi Omics technologies have recently been increasingly employed in the investigation of a wide range of diseases. These technologies serve as invaluable tools for identifying molecular profiles, uncovering novel biomarkers, delineating complex biochemical systems, and elucidating pathophysiological mechanisms [16]. These technologies have been employed in human osteoarthritis, prostate cancer, and nonalcoholic fatty liver disease and have been used to successfully identify molecular pathway alterations and potential therapeutic targets [13, 17, 18]. Integrated analysis of transcriptomics and metabolomics allows for a comprehensive understanding of biomolecule functions and regulatory mechanisms, revealing overall biological changes in an organism [17].

In the present study, our goal is to perform a comprehensive multi‐omic analysis to reveal pathological processes that could facilitate potential novel interventions for IDD. Thus, we conducted a metabolomics analysis of human IDD to identify distinct metabolic profile changes specific to senescent NP cells. Then, a subsequent transcriptomic analysis was carried out to pinpoint differential pathways that are indicative of senescent NP cell‐specific metabolic alterations. Finally, we integrated pathological phenotypes with transcriptomic and metabolomic findings to provide deeper insight into the pathomechanism of IDD. The outcomes of this integration can be utilized to generate new hypotheses and select candidate molecules for subsequent functional validation of therapeutic targets.

## 2 Methods and Materials

### 2.1 Patient Samples and NP Cells Culture

The ethics committee of our institution approved this study, and written informed consent was obtained from each enrolled participant. Prior to surgery, all patients underwent a routine MRI scan of the lumbar spine. The early stage of disc degeneration was defined as Pfirrmann classification I–II grade, while the advanced stage was defined as grades III–V [5]. The NP samples were collected from patients with IDD who underwent discectomy in our center. The surgical indications were specified as follows: [1] progressive neurologic deficits such as cauda equina syndrome or progressive motor weakness; or severe discogenic pain; [2] symptoms persisting for more than 3 months without response to conservative treatment. Exclusion criteria encompassed ankylosing spondylitis, diffuse idiopathic skeletal hyperostosis, and lumbar spinal infection.

The NP tissues were collected from patients operated on at our center. The NP tissues were fragmented and enzymatically digested in 0.2% type II collagenase (Gibco, USA) for 4 h at 37°C. After filtration and double washing with phosphate‐buffered saline (PBS), the suspension was centrifuged, and the isolated cells were cultured in Dulbecco's modified Eagle medium (DMEM) supplemented with 10% fetal bovine serum (FBS), 100 U/mL penicillin, and 100 mg/mL streptomycin in a 5% CO_2_ incubator at 37°C. Moreover, the cells were employed for IF staining in order to ascertain the expression of collagen II, thereby validating the identity of the cultured cells as NP cells [18]. The culture medium was refreshed every 3 days, and cells from the second or third passage were used for subsequent experiments.

### 2.2 Transmission Electron Microscopy (TEM)

The human NP cells were fixed overnight in a 2.5% glutaraldehyde solution, and subsequently treated with 1% osmium tetroxide at a temperature of 4°C for 1 h. The cells were subjected to a series of dehydration steps using ethanol concentrations in a graded manner, starting from 30% and progressing to 50%, 70%, and finally reaching 90%. Each dehydration step lasted for a duration of 30 min. Subsequently, the cells were further dehydrated three times with pure ethanol, each time lasting for 30 min. These samples were subjected to treatment with an embedding medium containing propylene oxide. Ultrathin sections of 70 nm were cut using an LKB‐V ultramicrotome machine (LKB, Sweden) and collected on copper grids. The ultrathin sections were subjected to staining with lead citrate for a duration of 10 min at room temperature, followed by three washes with deionized double‐distilled water. Subsequently, the sections were stained with uranyl acetate for 30 min at room temperature and then washed again three times with deionized double‐distilled water. The images were obtained in a JEM‐1400 transmission electron microscope system (JEOL, Japan).

### 2.3 Immunofluorescence Staining (IF)

The NP cell cultured in confocal dishes was fixed in 4% paraformaldehyde for 20 min, followed by two rinses with PBS. After permeabilization with 0.1% Triton X‐100 for 15 min, the samples were blocked with 2% goat serum for 30 min. Subsequently, the samples were incubated overnight at 4°C with a primary antibody, and Alexa Fluor‐594 or Alexa Fluor‐488 was used as the secondary antibody at room temperature for 1 h. The primary antibodies used were as follows: GRP78 (Servicebio biology, China; 1:200 dilution), CHOP (CST, USA; 1:150 dilution), TOMM20 (Servicebio biology, China; 1:200 dilution), P53 (Servicebio biology, China; 1:200 dilution), P21 (Servicebio biology, China; 1:150 dilution). The nucleus was labeled with DAPI for 5 min. Finally, the fluorescent images were captured using a confocal microscope.

### 2.4 Western Blot Analysis (WB)

Protein extraction was performed using RIPA Lysis Buffer (Beyotime, China) containing protease inhibitors. The cells were washed with PBS 3 times, then collected with RIPA and sonicated for 15 s to disrupt the cells, and the tube was then placed on ice for 30 min. The mixture was centrifuged at 12 000 rpm for 20 min at 4°C and the supernatant was collected. The concentration of protein was measured using the BCA protein concentration assay kit (Beyotime, China). Subsequently, the proteins were separated by sodium dodecyl sulfate polyacrylamide gel electrophoresis (SDS‐PAGE) and transferred onto a PVDF membrane. After blocking with 5% skim milk for 1 h, the membranes were incubated overnight at 4°C with the corresponding primary antibodies: GRP78 (Servicebio biology, China; 1:500 dilution), CHOP (CST, USA; 1:500 dilution), TOMM20 (Servicebio biology, China; 1:1000 dilution), P53 (Servicebio biology, China; 1:1000 dilution), P21 (Servicebio biology, China; 1:500 dilution), CPT1A (Proteintech, China; 1:1000, dilution), GAPDH (Servicebio biology, China; 1:2000 dilution). Following this, horseradish peroxidase (HRP)‐conjugated secondary antibodies were applied and the bands were visualized using an ECL reagent. GAPDH protein levels were used as loading controls. Protein bands were quantified by densitometry using ImageJ software (version 1.50).

### 2.5 Free Fatty Acid Measurements

PA content was determined using a free fatty acid assay kit (Solarbio, China). The cell samples were homogenized in the lysis buffer provided by the kit, followed by centrifugation to collect the supernatant for further analysis. Subsequently, the extract was thoroughly mixed with an organic solvent mixture of n‐heptane, methyl alcohol, and chloroform (in a ratio of 24:1:25) along with CuSO4 solution. After centrifugation, the oil phase (upper layer) was separated from the water phase (lower layer). The resulting supernatant was then pipetted for measuring copper salt levels that had bound to free fatty acids. The BCA assay (Beyotime, China) was employed to determine total protein concentrations for the purpose of normalizing free fatty acid content.

### 2.6 JC‐1 Staining

The mitochondrial membrane potential (MMP) changes were evaluated using the JC‐1 fluorescent probe (Beyotime, China). JC‐1 stock solution was diluted 200‐fold by adding 50 μL of JC‐1 to 8 mL of ultrapure water, ensuring complete dissolution and thorough mixing. Next, 2 mL of JC‐1 staining buffer (5X) was added and mixed thoroughly to prepare the working solution for JC‐1 staining. The samples were then incubated with this working solution at 37°C for 20 min. After two washes with a buffer solution, fluorescence images were acquired using a confocal microscope. Mitochondria with higher potential were stained red, while those with lower potential were stained green, indicating early‐stage apoptosis. MMP was quantified as the ratio of red to green fluorescence intensities.

### 2.7 Bodipy Staining

BODIPY is a commonly used fluorescent stain for labeling lipid droplets. Fixed cells were incubated with a BODIPY (Cayman Chemical, USA) solution at 37°C in the dark for 30 min. Subsequently, the samples were washed twice with PBS and then labeled with DAPI for 10 min. Finally, the samples were observed using a confocal microscope to capture images. The lipid droplets were quantified by Bodipy staining, where the fluorescent dots indicate the presence of lipid droplets.

### 2.8 ROS Staining

ROS staining was performed using a ROS staining kit (Beyotime, China). DCFH‐DA was diluted in serum‐free culture medium at a ratio of 1:1000 to achieve a final concentration of 10 micromolar for the preparation of the ROS solution. The samples were seeded in confocal dishes and incubated with a ROS solution for 15 min at 37°C. After being washed three times with serum‐free cell culture medium, the images were acquired using a confocal microscope. The ROS level generated in cells is assessed by quantifying the green fluorescence intensity upon irradiation with a 488 nm laser. The signal is measured and analyzed using ImageJ software (version 1.50) to determine the fluorescence intensity.

### 2.9 Endoplasm Reticulum and Mitochondrial Labeling

Cells were seeded in coronal dishes and treated as required in each experiment. For each experiment, NP cells were incubated in the absence of palmitate, the presence of palmitate (0.2 mM), the presence of palmitate (0.2 mM) + PC (3 mg/mL), or the presence of palmitate (0.2 mM) + PC (3 mg/mL) + Tunicamycin (1 μg/mL). The endoplasmic reticulum was labeled with ER‐tracker green (Beyotime, China). Mitochondria were labeled with Mito‐Travker red (Beyotime, China). Cells were incubated with the working solution containing the desired fluorescent probes for 30 min at 37 °C. For ER and mitochondrial network staining, ER‐Tracker green and MitoTracker red were used. Finally, the fluorescent images were captured using a confocal microscope.

### 2.10 Cell Viability Assessment

The cell viability was assessed using the Cell Counting Kit‐8 (CCK‐8) methods (Beyotime, China) according to the manufacturer's recommendations. After seeding the samples in a 96‐well plate and applying the respective treatments, a 10 μL CCK‐8 solution was added to each well for a duration of 4 h. Subsequently, the absorbance at 450 nm was measured using a spectrophotometer. The optical density of untreated wells was set as 100%, and the percentage cell viability was determined in the test well by comparing the control optical density.

### 2.11 Endoplasm Reticulum and Mitochondrial PC Contents Evaluation

The PC content was measured using a PC colorimetric/fluorometric assay kit (BioVision, USA) following the manufacturer's instructions. In brief, the samples, PC assay and reaction buffer were added to 96‐well plates and incubated for 3 h in the dark. Using a microplate reader to measure the absorbance at 570 nm (OD value) can indirectly reflect the level of PC, and the results are presented as relative ratios of absorbance compared with the respective control or untreated cells. The ER fractions for evaluation of ER PC contents were purified using the Endoplasmic Reticulum Isolation Kit (Sigma‐Aldrich, USA) according to the recommended protocol [19]. A positive control utilizing calnexin was employed. The mitochondria fractions for evaluation of PC abundance were purified using the Mitochondria Isolation Kit (Solarbio, China) according to the manufacturer's instructions. The TOMM20 protein was utilized as an internal control for mitochondrial content normalization for mitochondrial PC contents evaluation [20].

### 2.12 Senescence β‐Galactosidase Staining

The senescence activity of the samples was evaluated using the SA‐β‐gal staining kit (Beyotime, China). The samples were seeded in a six‐well plate and fixed with 1 mL of β‐galactosidase stain fixing solution for 15 min at room temperature, followed by three washes with PBS. Subsequently, they were stained with 1 mL of working staining solution overnight at 37°C without CO_2_ supply. Finally, to prevent evaporation, the samples were sealed with plastic wrap. After staining, the samples exhibited a blue coloration, indicating an elevated SA‐β activity. The cells were subsequently examined under a microscope to observe the presence of blue coloring.

### 2.13 EDU Staining

The samples were seeded and cultured in confocal dishes, followed by performing the EDU assay according to the manufacturer's protocol using the BeyoClick EdU‐488 detection kit (Beyotime, China). After labeling for 12 h, the cells were fixed using a 4% paraformaldehyde solution and permeabilized with 0.5% Triton X‐100. Subsequently, the cells were incubated in the Click solution for a duration of 30 min and stained with DAPI. The samples were visualized using a confocal microscope.

### 2.14 Transcriptomic Sequencing

The NP cells were collected from 4 patients with early‐stage IDD and 4 patients with advanced‐stage IDD. Total RNA was extracted using the TRIzol RNA extraction reagent (Invitrogen, United States). The quality of the extracted RNAs was evaluated using a NanoDrop 2000 spectrophotometer manufactured by Thermo Scientific (USA). Furthermore, RNA integrity was assessed using the Agilent 2100 Bioanalyzer developed by Agilent Technologies (USA), and only samples with an RNA integrity number exceeding 9 were chosen for library preparation. Subsequently, libraries were generated using the TruSeq Stranded mRNA LT Sample Prep Kit provided by Illumina (USA). These libraries underwent sequencing on an Illumina HiSeq X 10 platform to generate paired‐end reads measuring 150 bp in length. To obtain high‐quality reads, raw data in fastq format were initially processed through Trimmomatic to eliminate poly‐N sequences and low‐quality reads. Finally, alignment of clean reads to the human reference genome was performed utilizing HISAT2 software.

The FPKM values for each gene were quantified using cufflinks, and the gene counts were obtained using htseq‐count. Differential expression analysis between the two groups was conducted using the DESeq (2012) R package. Genes exhibiting significant differential expression were determined based on a threshold of *p*‐value < 0.05 and fold change > 2 or fold change < 0.5. Hierarchical clustering analysis was performed to elucidate distinct expression patterns between the early‐stage and advanced‐stage groups. GO enrichment and KEGG pathway enrichment analyses of the differentially expressed genes (DEGs) were carried out using the R package (version 4.0.3) based on the hypergeometric distribution.

### 2.15 Metabolomic Sequencing

The NP cells were collected from the same patients for transcriptomics. Add 20 μL of internal standard (2‐chloro‐l‐phenylalanine in methanol, concentration: 0.3 mg/mL) and 400 μL of the extraction solvent composed of methanol/water (ratio: 4/1, v/v). Then, rapidly cool NP cells to −80°C for a duration of 2 min, grind at a frequency of 60 Hz for a period of 2 min, ultrasonicate at ambient temperature for 10 min following vortexing, and subsequently place at −20°C for an additional time frame of 30 min. After centrifugation, cautiously transfer a volume of 150 μL supernatant to a new tube and then proceed with drying using freeze concentration centrifugal dryer equipment. Store the resulting dried samples at −80°C until LC‐MS analysis can be conducted. To ensure quality control during analysis, combine aliquots from all individual samples to create a pooled sample.

The LC analysis was conducted using an ACQUITY UPLC HSS T3 column (100 mm × 2.1 mm, 1.8 μm) on an ACQUITY UHPLC system (Waters Corporation, USA) coupled with an AB TripleTOF 6600 plus system (AB SCIEX, MA) in both positive‐ion (ESI+) and negative‐ion (ESI−) electrospray ionization modes. The mobile phase consisted of solvents A: water (containing 0.1% formic acid, v/v) and B: acetonitrile (containing 0.1% formic acid, v/v). Separation was achieved using the following gradient program: at the start of the run, B concentration was set to 5%; at 2 min, B concentration increased to 20%; at 4 min, B concentration further increased to 60%; at 11 min, B concentration reached its maximum of 100%, which was maintained until Minute 13; then it decreased back to initial conditions within half a minute and remained constant for another minute before starting the next run. The flow rate during analysis was set at a constant value of mL/min while maintaining the column temperature at °C throughout the experiment duration. The injection volume used for each sample was μL. The samples were stored under refrigeration conditions(4°C). Data acquisition involved full scan mode from m/z range of 70–1000 in combination with information‐dependent acquisition mode. MS parameters included ion source temperature settings of 550°C (+) and 550°C (−), ion spray voltage values of 5500 V (+) and 4500 V(−), curtain gas pressure at 35 PSI, and declustering potential settings at 100 V (+) (−) with collision energy values of 10 eV(+) (−). The interface heater temperature settings were 550°C (+) and 600°C(−). For IDA analysis, the mass‐to‐charge ratio(m/z) value ranged from 25 to 1000, and collision energy was fixed at 30 eV for identification purposes.

The LC‐MS raw data obtained were extracted and processed using Progenesis QI software (Waters Corporation, Milford, MA, USA). The parameters were set as follows: precursor tolerance of 5 ppm, fragment tolerance of 10 ppm, and production threshold of 5%. The resulting matrix was utilized to construct three‐dimensional data sets comprising retention time, mass‐to‐charge ratio (m/z), and normalized ion intensities. The matrix was further reduced by eliminating any peaks with missing values (ion intensity = 0) in over 50% of the samples. Metabolites were identified using progenesis QI (Waters Corporation, Milford, USA) Data Processing Software based on public databases such as http://www.hmdb.ca/; http://www.lipidmaps.org/ and self‐built databases (Ouyi Corporation, Shanghai, China). Both positive and negative data were combined and imported into R software. Orthogonal partial least‐squares‐discriminant analysis (OPLS‐DA) was conducted to visualize the differences between the groups. Differential metabolites were selected from the OPLS‐DA model using a combination of VIP value > 1 and *p* ≤ 0.05 through a two‐tailed Student's *t*‐test on the normalized peak areas. The differential metabolites were annotated using the KEGG database and mapped to KEGG pathways.

### 2.16 Lipidomics

Lipid extraction and lipid detection utilizing mass spectrometry were performed as follows: 200 μL of cold water and 20 μL of a lipid standard internal mixture were added to NP cells for the purpose of lipid extraction. Subsequently, the samples were homogenized at 4°C using an MP homogenizer. Following homogenization, 800 μL of cold methyl tert‐butyl ether and 240 μL of methanol were added to the samples, which were then vortexed for 30 s and sonicated at 4°C for 20 min. The samples were then allowed to stand for 30 min before being centrifuged at 14 000 g for 15 min at 10°C to isolate the lipids. The upper organic layer containing the lipids was then dried using a vacuum centrifuge. The dried samples were resuspended in 200 μL of a solution composed of isopropanol and acetonitrile in a 9:1 (v/v) ratio and utilized for lipidomic analysis.

In the context of lipidomic analysis, lipid extracts were subjected to LC–MS analysis. Subsequently, LC‐MS/MS analysis was carried out utilizing a Q Exactive plus mass spectrometer (Thermo Scientific) in conjunction with a UHPLC Nexera LC‐30A (Shimadzu). In summary, the lipids were fractionated on a Waters ACQUITY PREMIER CSH C18 Column (1.7 μm × 2.1 × 100 mm) under specified chromatographic parameters: mobile phase A comprised of acetonitrile: water in a ratio of 6:4 (v/v), while mobile phase B consisted of acetonitrile: isopropanol in a ratio of 1:9 (v/v). The flow rate was maintained at 300 μL/min, and the column was heated to a temperature of 45°C. MS detection and analysis were conducted using electrospray ionization (ESI) in both positive and negative ion modes. Full‐scan spectra were acquired within mass‐to‐charge ratio (m/z) ranges of 200–1800 and 250–1800, respectively, for positive and negative ion modes. The identification, extraction, alignment, and quantification of lipids were accomplished with LipidSearch software version 4.1 (Thermo Scientific).

### 2.17 Magnetic Resonance Spectroscopy (MRS) Analysis

A group of healthy volunteers and patients with IDD were selected for this study. All participants were informed, and informed consent was obtained. The participants information for MRS analysis has been uploaded in the Table S1. In order to focus on regions of interest in the sagittal, coronal, and axial planes, a single‐voxel spectroscopy was assigned by the scan operator for the intervertebral space scan regions, which specifically included the disc nucleus while excluding the vertebral body. Lumbar spinal MRI was conducted following routine procedures, with MRS data collected during a secondary MRI session after completion of clinical imaging. Following precise localization of the MRS voxel, proton MRS scans were performed; the time interval between two successive radio frequency pulses (Time of Repetition, TR) was set as 1000 ms, and the duration between the application of a radio frequency pulse and its corresponding echo (Time of Echo, TE) was set as 37 ms. It is important to note that the collected MRS data were not utilized for surgical purposes. Quantitative results were expressed as area under the lipids peak for IDD normalized area under the lipids peak for healthy control.

### 2.18 Animal Experiment

All animal studies were approved by the institutional animal care and use committee of the first affiliated hospital of USTC. Three‐month‐old male Sprague–Dawley rats were used for the animal experiments according to methods as reported previously [21, 22]. The rats were randomly divided into four groups, each consisting of five rats: Control group, IDD group with needle puncture, PA group with PA injection, and PA + PC group with PA and PC injection. Following anesthesia, the rat IDD model was established by puncturing the tail disc at the Co8‐Co9 level using a 20‐gauge needle inserted to a depth of approximately 5 mm. The needle was then passed vertically through the tail disc, contralateral skin, and rotated 180° for 15 s. After establishing the IDD model in the PA + PC group, an injection of PC solution at a dosage of PC (2 mg/mL, 3 μL) was administered into the indicated disc. The PA + PC group received an equal dosage mixture solution containing both PA and PC. In contrast, the IDD group received an intradiscal injection of saline solution into their discs. These drug injections were performed once weekly for a total duration of 4 weeks.

### 2.19 Histology and Immunohistochemical Staining

For human NP tissues, the samples were fixed in a 4% paraformaldehyde solution. Subsequently, the NP tissues underwent dehydration and embedding in paraffin, followed by slicing into 5 μm sections. Slides of each sample were stained with hematoxylin and eosin (HE), Masson, and Alcian blue.

For the rat tail, the Co8‐Co9 level, including the whole upper endplate of the head‐end vertebra and the lower endplate of the tail‐end vertebra, was harvested and fixed in a 4% paraformaldehyde solution overnight at 4°C, decalcified using a 10% EDTA solution for 1 month. Subsequently, the samples underwent dehydration and embedding in paraffin. The paraffin blocks were cut into 5 μm slices in the coronal plane. The slices of rat tail were stained with HE staining, safranin O/fast green (S—O) staining, and Alcian blue.

The immunohistochemical (IHC) staining was performed for human NP tissues and rat tail. After deparaffinization and rehydration, the sections were boiled in 0.01 mol/L citrate buffer (pH 6.0) for 15 min in a microwave oven. Then, blocking with goat albumin for 30 min, the samples were incubated overnight at 4°C with the primary antibody. The primary antibodies information is as follows: anti‐GRP78 (Servicebio biology, China; 1:300 dilution), anti‐CHOP (CST, USA; 1:400 dilution), anti‐P53 (Servicebio biology, China; 1:300 dilution), anti‐P21 (Servicebio biology, China; 1:300 dilution). Subsequently, a secondary antibody was applied to the samples for 1 h at room temperature, followed by hematoxylin staining. Finally, the slices were examined under a confocal microscope to capture images.

### 2.20 Statistical Analysis

The data were presented as the mean ± standard deviation (SD), and a minimum of three independent experiments were conducted. The statistical differences between two groups were determined using Student's t‐test for normally distributed data, or a Mann–Whitney U test for non‐normally distributed data. For multi‐group comparisons, anova was applied for normally distributed data or the Kruskal‐Wallis test for non‐normally distributed data. Correlation between two quantitative variables was calculated using Spearman's rank correlation test. Statistical significance was determined using GraphPad Prism software, with the threshold set at *p < 0.05, **p < 0.01, and ***p < 0.0001.

## 3 Results

### 3.1 Lipotoxicity Is Associated With Disc Degeneration in Human IDD

**FIGURE 1:**
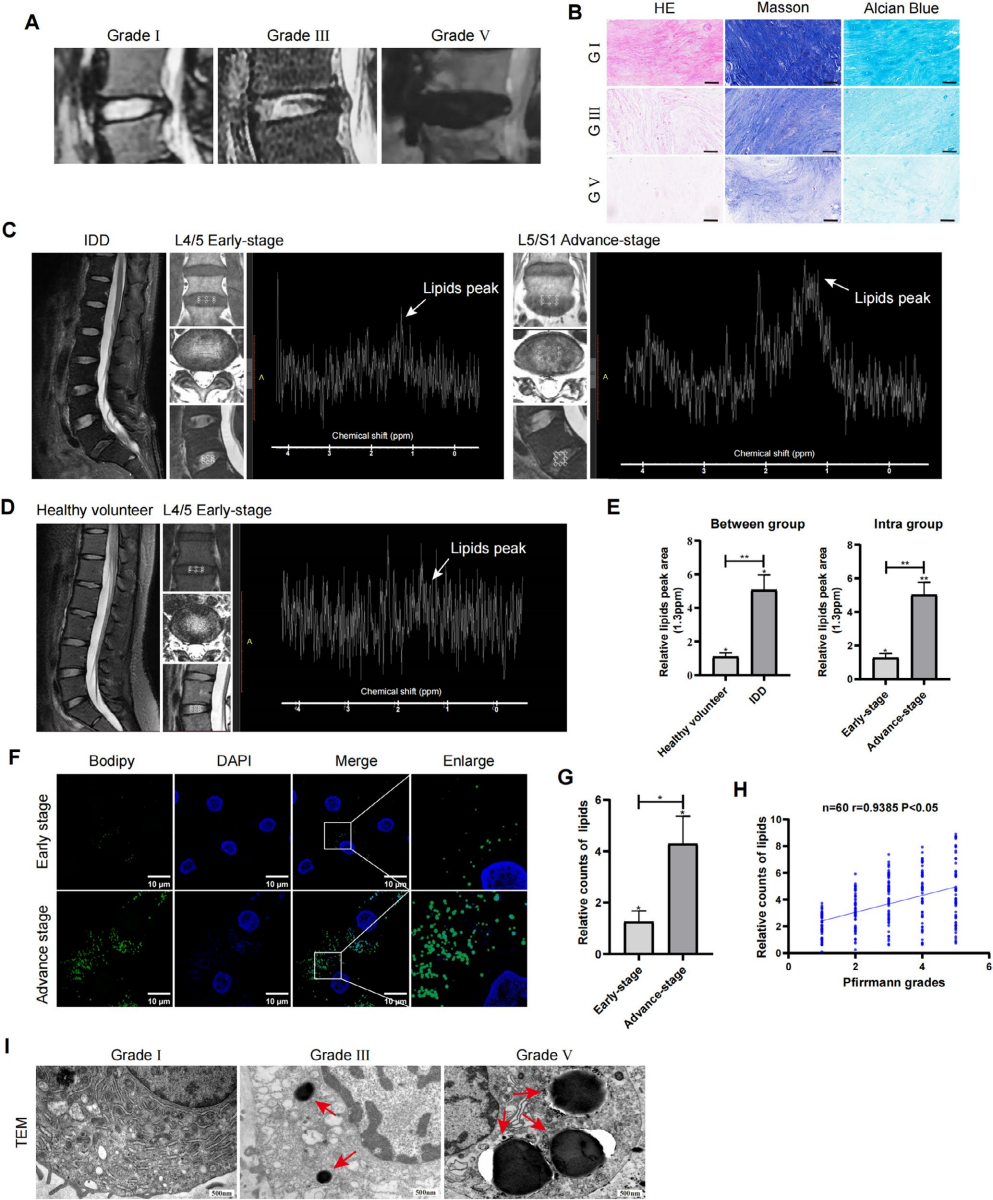
Increased lipotoxicity were detected in IDD. (A) Representative T2‐weighted MR images of the intervertebral disc in humans (Grade I, Grade III, and Grade V). (B) The HE, Masson, and Alcian blue staining were performed on NP from human intervertebral discs at early‐ and advanced‐stages. Scale bar:50 μm. (C–D) MRS was conducted on normal and degenerated discs. The metabolic spectrum changes detected by MRS indicated the presence of abnormal lipids peaking at 1.3 ppm in patients with IDD. (E) Statistical result of the relative area under the lipid peak between early‐ and advanced‐stage groups. *n* = 6.***p < 0.001. For each IDD patient, the spectrum demonstrated a higher lipid peak for degenerated disc (advanced‐stage) and a relatively lower lipid peak for healthy disc (early‐stage). *n* = 6.***p < 0.001. (F, G) Bodipy staining of NP cells for lipid droplets was performed at early‐ and advanced‐stages. Scale bar:10 μm. Statistical result of the relative lipid droplet amount between early‐ and advanced‐stage groups. *n* = 6.***p < 0.001. (H) The lipid contents were positively correlated with the Pfirrmann scores (*r*=, *p* <). (I) Lipid droplets were observed using TEM in different grades of NP samples.

The degree of IDD was evaluated based on the results of the T2‐weighted MR images using the Pfirrmann grading system. Various signal patterns were observed in degenerated intervertebral discs of different degrees (Figure 1A). Histological evaluation of NP tissues from early‐ and advanced‐stage IDD patients was performed via HE, Masson, and Alcian blue staining (Figure 1B). MRS analysis revealed a greater peak at approximately 1.3 ppm in patients with IDD, and the area under the lipid peak was significantly greater in advanced‐stage cases than in early‐stage cases, indicating lipid accumulation (Figure 1C,D). The lipid content in normal discs was lower than that in degenerated discs (Figure 1E). Bodipy staining revealed the accumulation of lipid droplets in NP cells from cases of advanced‐stage and revealed a greater relative number of lipids in advanced‐stage cases than in early‐stage cases (Figure 1F,G). Furthermore, there was a positive correlation between relative lipid counts and the degree of disc degeneration (Figure 1H). TEM observations of the NP cells revealed ultrastructural changes, including the accumulation of lipid droplets within degenerated nucleus pulposus cells (Figure 1I). These findings suggest that there is an abnormal accumulation of lipid droplets in patients with IDD.

### 3.2 Metabolic Profile Alterations During the Course of IDD

**FIGURE 2:**
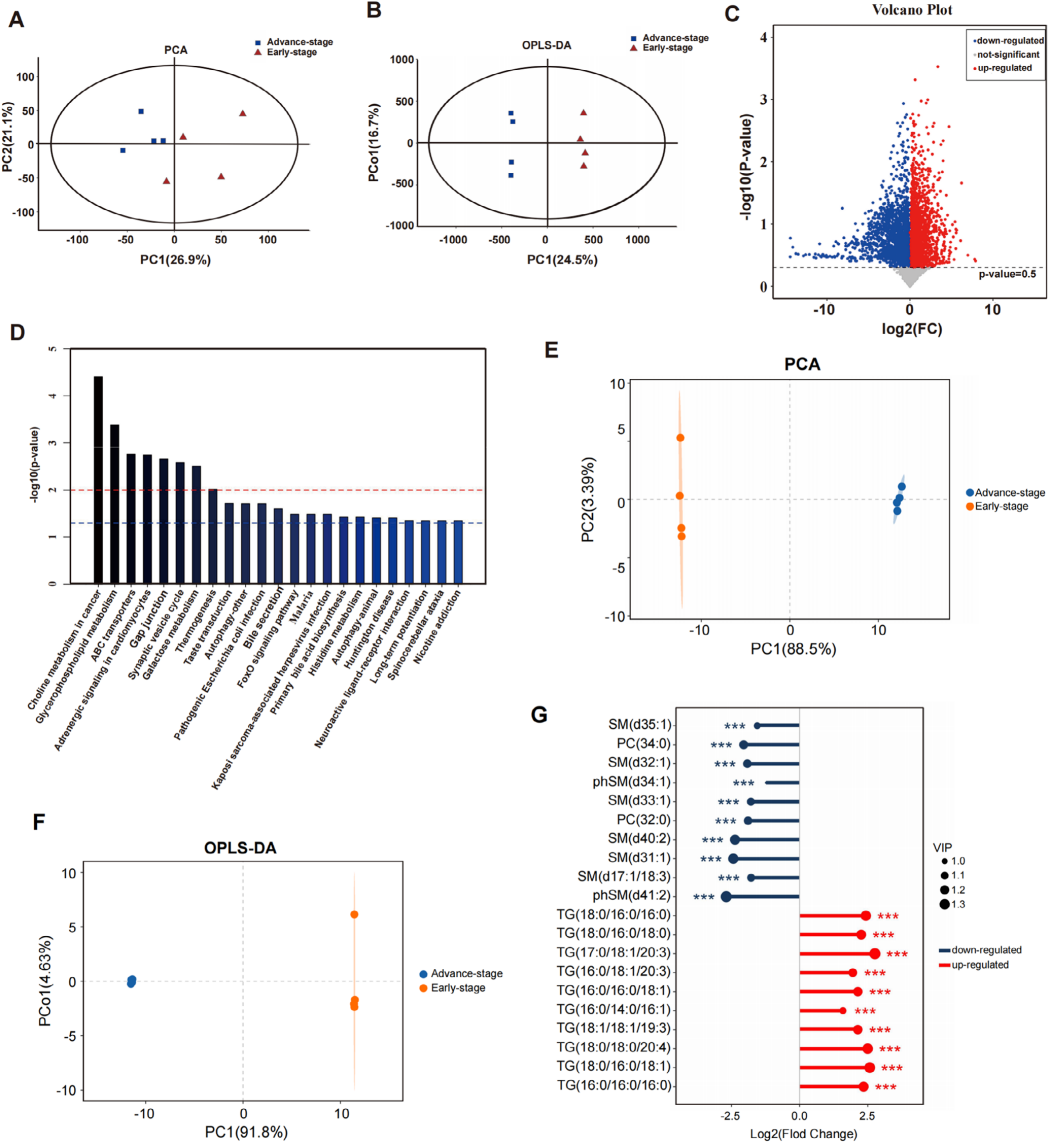
Metabolic profile changes during IDD. (A–B) Principle Component Analysis (PCA) and Orthogonal partial least squares‐discriminant analysis (OPLS‐DA) score plots for differentiating the metabolites in each group, the results showing an obvious separation between early‐stage and advanced‐stage. (C) Volcano‐plot showing the differential metabolites. (D) Kegg enrichment map of metabolic pathways. (E–F) Lipidomics were performed for NP cells; PAC and OPLS‐DA indicated a clear separation between the two groups. (G) The lolipopmap revealed the level of TGs was significantly increased in advanced‐stage.

To validate the MRS findings, LC/MS‐based untargeted metabolomics analysis was performed. principal component analysis (PCA) and orthogonal partial least squares discriminant analysis (OPLS‐DA) models were utilized to examine the metabolites in the NP samples, revealing distinct differences between early‐ and advanced‐stage samples (Figure 2A,B). Differentially abundant metabolites were analyzed via volcano plots and KEGG pathway enrichment approaches, which revealed significant alterations in the “glycerophospholipid metabolism (Has00564)” pathway (Figure 2C,D). Considering that MRS analysis revealed abnormal lipid peaks in advanced‐stage IDD samples, our focus shifted towards lipids. Lipidomics was performed on NP cells from early‐ and advanced‐stage IDD patients, and PAC and OPLS‐DA clearly distinguished between the two groups (Figure 2E,F). Lolipopmap revealed that the level of TGs significantly increased in the advanced‐stage (Figure 2G).

### 3.3 Combined Transcriptomics and Metabolomics Analysis Revealed a Decrease in PC Levels in IDD Patients

**FIGURE 3:**
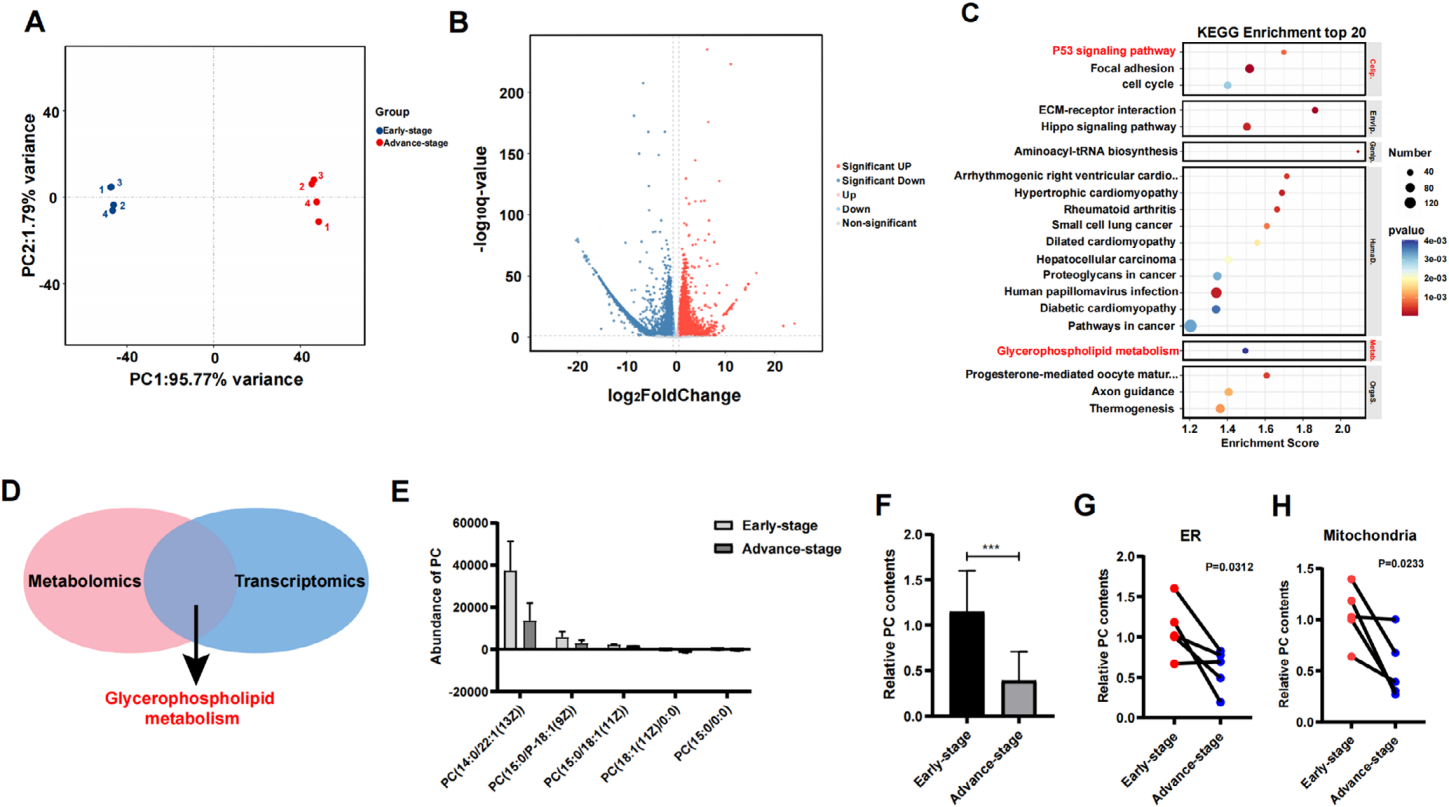
Complementary transcriptomics unveiled aberrant pathway. (A) PCA shows a significant difference in transcriptomics between the two groups. (B) Volcano plots showing the results of the differential gene expression analysis. (C) Top20 of KEGG signaling pathway enrichment of DEGs. (D) The integration of metabolomics and transcriptomics analysis revealed a significant alteration in the “glycerophospholipid metabolism” pathway. (E) Abundance of PC with different PC subtypes metabolites in the early stage and advanced stage. (F) The relative PC levels in NP cells were measured in early‐ and advanced‐stage patients, *n* = 30 for early stage and 30 for advanced stage. ***p < 0.001. (G ~ H) The relative mitochondria and endoplasmic reticulum PC abundance in pairs of samples in patients with different IDD severities from each patient. The relative contents of PC were measured about the ER and mitochondria after purification.

Transcriptomic data are employed to elucidate the mechanisms underlying the disruption of metabolic pathways and the initiation of disease‐associated phenotypes. NP samples were collected from four patients with early‐stage IDD and 4 patients with advanced‐stage IDD for transcriptome profiling. The PCA plot revealed two distinct groups (Figure 3A). The transcriptomic analysis revealed that a total of 5182 genes were downregulated and that 2817 genes were upregulated between the early and advanced stages (Figure 3B). The KEGG pathway enrichment analysis of the DEGs revealed that the top 20 enriched KEGG terms are depicted in Figure 3C. Notably, “glycerophospholipid metabolism” emerged as a significant finding. Specifically, the downregulated subset of DEGs also exhibited significant enrichment in the pathways “Fatty acid biosynthesis,” “Fatty acid metabolism,” and “glycerophospholipid metabolism” according to the KEGG analysis (Figure S1A,B). In addition, GSEA revealed abnormal mitochondrial behavior in advanced‐stage IDD, suggesting impaired mitochondrial oxidation capacity (Figure S1C).

The integrated analysis of transcriptomic and metabolomic data revealed an aberrant “glycerophospholipid metabolism” pathway, in which certain subtypes of PC within the glycerophospholipid metabolism pathway decreased in concentration, specifically PC(14:0/22:1(13Z)), PC(15:0/P‐18:1(9Z)) and PC(15:0/18:1(11Z)). Conversely, other subtypes, such as PC(18:1(11Z)/0:0) and PC(15:0/0:0), increased in concentration during the advanced stage. However, it is important to note that the subtypes of PC whose concentrations increased in the advanced stage were present at significantly minuscule abundance (Figure 3D,E). In the validation set, NP cells from 30 early‐stage and 30 advanced‐stage IDD patients were collected to determine the PC abundance. The total PC level in the advanced stage was significantly lower than that in early stage (Figure 3F). Additionally, we also performed a comparison between pairs of discs with different IDD severities from each patient. All five patients had an early‐stage segment and an advanced‐stage segment; the metabolite PC was significantly downregulated across the mitochondria and the endoplasmic reticulum (Figure 3G,H).

### 3.4 PC Protected ER and Mitochondrial Function in the Presence of PA

**FIGURE 4:**
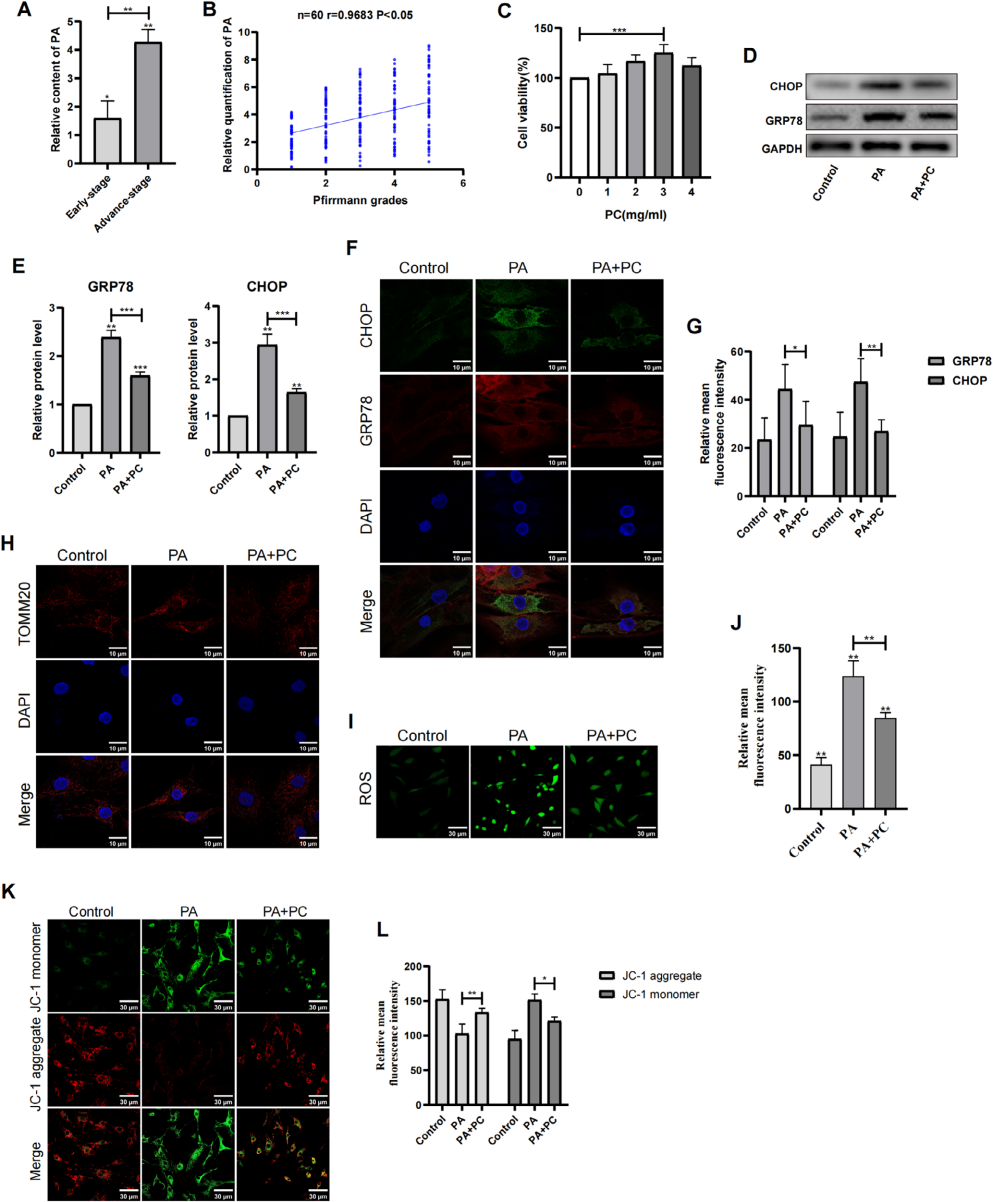
PC protected the function of ER and mitochondria under PA condition. (A) The PA levels of NP cells were determined in 30 early‐stage and 30 advanced‐stage IDD patients; (B) Single‐factor linear regression analysis of the relationship between the relative PA contents and Pfirrmann grade; (C) NP cells were incubated with PC of different concentrations and CCK‐8 was used to detect the activity. (D–E) WB and quantification showed the expression of ER stress marker GRP78 and CHOP in different groups (Control, PA, PA + PC). *n* = 3. ***p < 0.001. (F–G) IF staining of GRP78 and CHOP. Scale bar: 10 μm. Statistical result of the relative mean fluorescence intensity on the right. *n* = 6. ***p < 0.001. (H) IF staining of mitochondrial‐related protein TOMM20 in human NP cells. Scale bar: 10 μm. (I–J) The ROS staining and comparison of the relative mean fluorescence intensity between different groups. Scale bar: 30 μm. *n* = 3. ***p < 0.001. (K–L) The JC‐1 staining and comparison of the relative mean fluorescence intensity between different groups. Scale bar: 30 μm. *n* = 3. ***p < 0.001.

The PA level in the advanced‐stage NP cells was significantly higher than that in early‐stage NP cells (Figure 4A). In addition, the PA content was positively correlated with disc degeneration (Figure 4B). PA is one of the most common free fatty acids in the body, but there is limited information available regarding the potential toxic effects of PA on NP cells. Our findings revealed that PA results in ER stress, mitochondrial damage, increased ROS levels, decreased MMP, and resulted in lipid droplet accumulation in NP cells (Figure S2A–L). The findings from the multiomics analysis and functional validation indicate the involvement of the PA and PC in nucleus pulposus lipotoxicity.

NP viability was significantly enhanced by PC treatment after 72 h at concentrations ranging from 1 to 4 mg/mL (Figure 4C). Notably, the most pronounced protective effect was observed at a concentration of 3 mg/mL, which was chosen for subsequent experiments. Following PA intervention, PC treatment was administered to NP cells. The results of WB and IF staining demonstrated a decrease in the expression of the ER stress markers GRP78 and CHOP following PC treatment (Figure 4D–G). Moreover, the assessment of mitochondrial morphology, ROS levels, and the MMP indicated that PC effectively preserved mitochondrial function in the presence of PA (Figure 4H–L).

### 3.5 PC Facilitates the Interaction of the ER With Mitochondria

**FIGURE 5:**
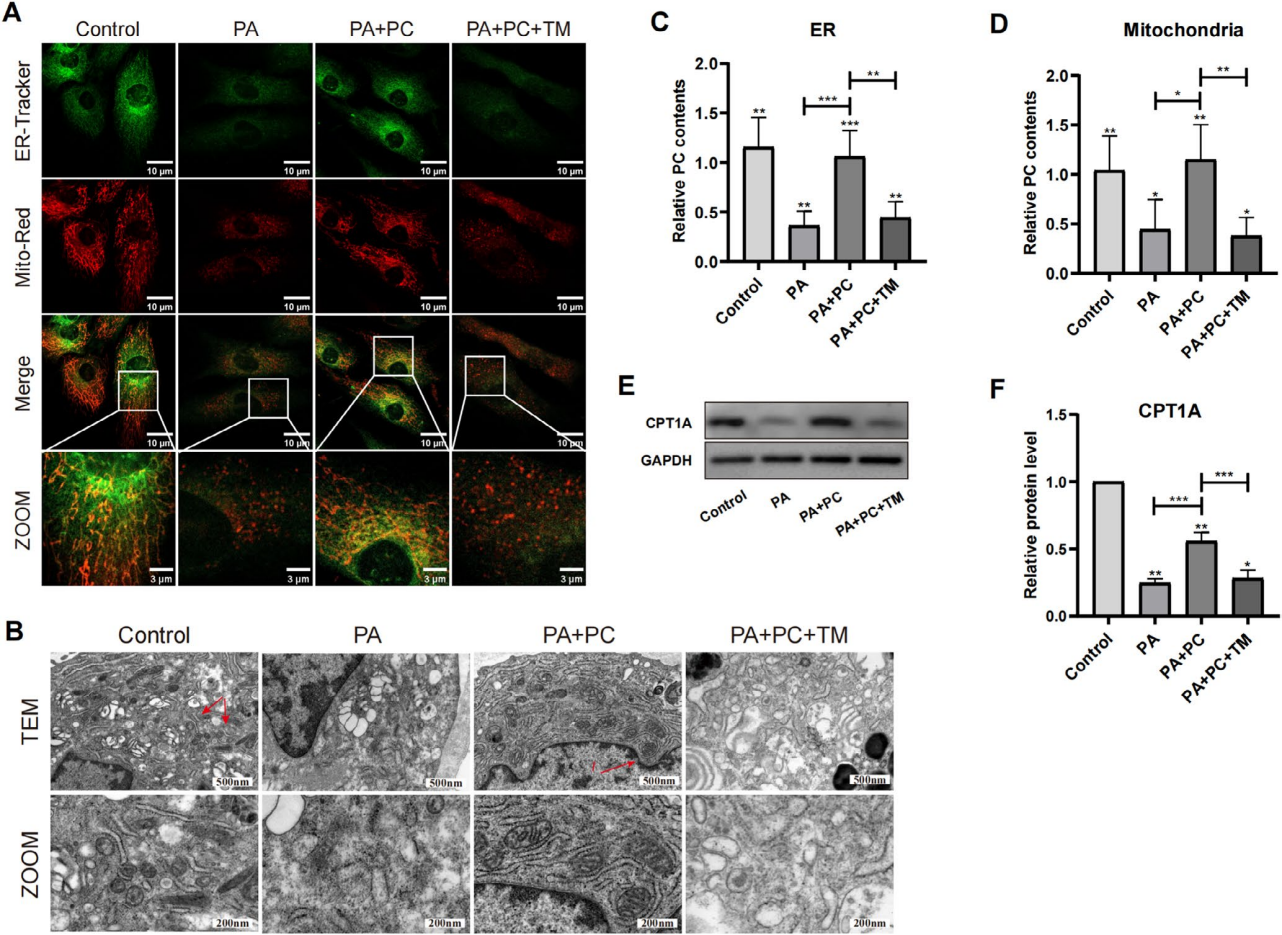
PC facilitating the interaction between ER and mitochondria. (A) Representative confocal images illustrating the contacts between ER and mitochondria in human NP cells. Scale bar: 10 μm and 3 μm (ZOOM). (B) The contacts between ER and mitochondria were detected by TEM. Scale bar: 500 nm and 200 nm (ZOOM). (C–D) The relative PC contents of ER and mitochondria in NP cells after purification were measured. Comparison of the relative PC contents between different groups. *n* = 3. ***p < 0.001. (E–F) The expression levels of fatty acid oxidation relative protein CPT1A were analyzed by WB in NP cells. *n* = 3. ***p < 0.001.

The physical communication between the endoplasmic reticulum (ER) and mitochondria plays a pivotal role in determining lipid transfer and mitochondrial function. The findings of our study demonstrate that PA impairs the interactions between the endoplasmic reticulum and mitochondria and that PC facilitates the interaction between the ER and mitochondria under PA intervention (Figure 5A,B). The relative content of PC was detected by the purification of ER and mitochondria. PA treatment decreased the PC content in the ER and mitochondria, but after PC treatment, the relative content of PC increased (Figure 5C,D). CPT1A, the rate‐limiting enzyme of fatty acid oxidation, can promote the uptake of fatty acids and mitochondrial β‐oxidation, reflecting the mitochondrial oxidation capacity. PC treatment improved the expression of CPT1A (Figure 5E,F). These results showed that PC facilitated the interaction between the ER and mitochondria and improved the mitochondrial PC content and fatty acid oxidation.

### 3.6 PC Alleviated PA‐Induced Lipid Droplet Accumulation and NP Cell Senescence in an ER‐ and Mitochondrial‐Dependent Manner

**FIGURE 6:**
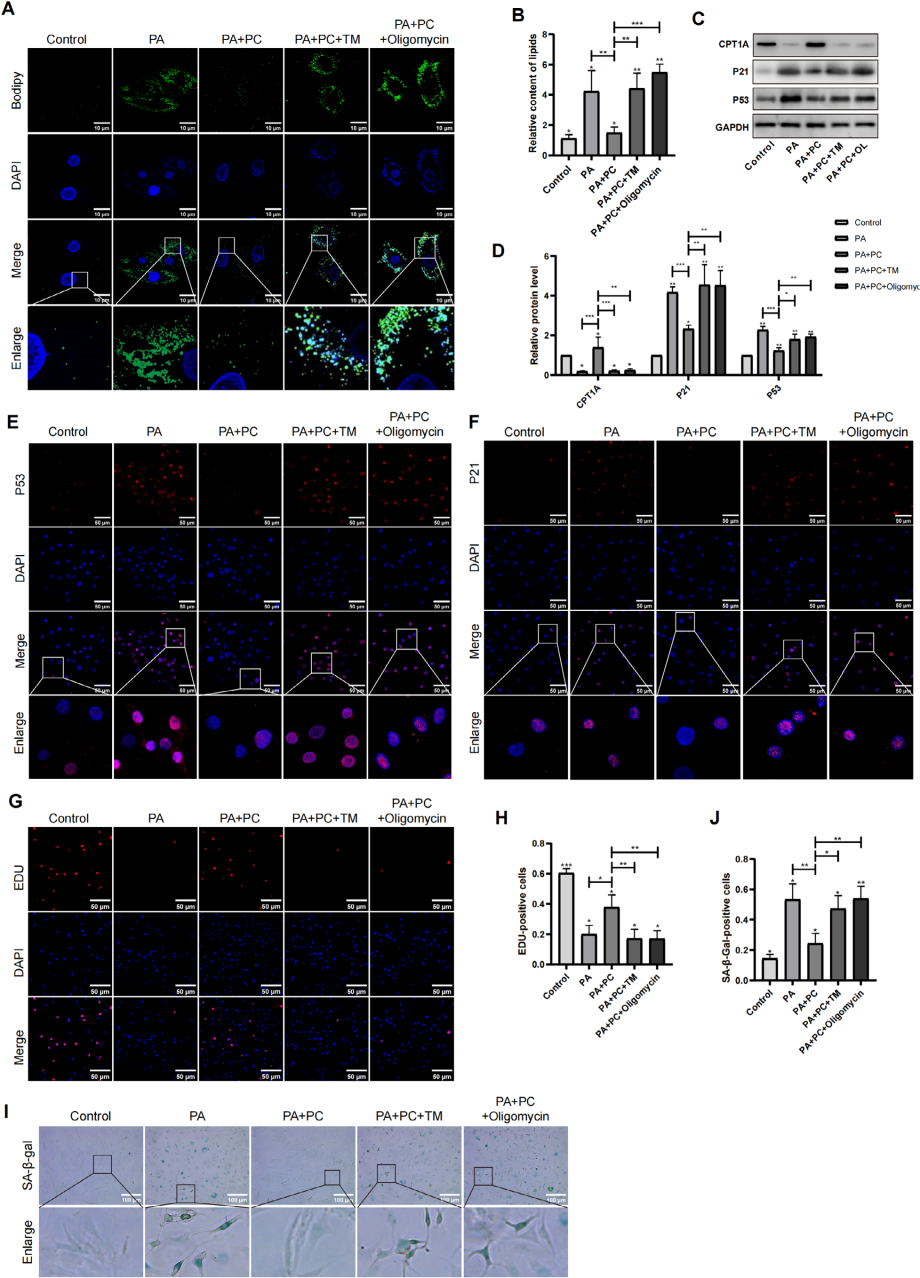
PC alleviated lipid droplet accumulation and cell senescence in both ER and mitochondria dependent manner. NP cells were incubated with or without the ER stress inducer TM and the mitochondrial respiratory inhibitor OL. (A, B) Bodipy staining and statistical analysis for lipid droplets in NP cells. Scale bar: 10 μm. *n* = 3. ***p < 0.001. (C, D) WB analysis of CPT1A and P53, P21 expression levels in human NP cells. *n* = 3. ***p < 0.001. (E, F) IF staining of P53 and P21 in human NP cells. Scale bar: 50 μm. (G‐H) EDU staining detected the proliferation of human NP cells. Scale bar: 50 μm. The positive EDU cells were shown in the chart. *n* = 3. ***p < 0.001. (I, J) Representative Senescence β‐Galactosidase staining of NP cells. Scale bar: 100 μm. The positive SA‐β‐Gal cells were shown in the chart. *n* = 3. ***p < 0.001.

The present study further investigated the potential protective effects of PC on NP cells against lipid droplet accumulation and senescence induced by PA. After PC treatment, the NP cells were cocultured with either the ER stress inducer tunicamycin (TM) or the mitochondrial respiratory inhibitor oligomycin (OL) to block the ER and mitochondrial pathways, respectively. The BODIPY staining results revealed that PC significantly attenuated the accumulation of lipid droplets after PA treatment, whereas treatment with TM or OL blocked the effect of PC (Figure 6A,B). In addition, PC reduced the levels of P53 and P21 and increased the level of CPT1A, but these effects were blocked in the presence of TM or OL (Figure 6C,D). Cell senescence was also examined via P53 and P21 IF staining, senescence β‐galactosidase, and EDU assays, which revealed that PC significantly reduced the level of cell senescence, but treatment with TM or OL impeded the protective effect of PC (Figure 6E–J). These results demonstrated that PC could attenuate PA‐induced lipid droplet accumulation and cell senescence through the interaction of the ER and mitochondria.

### 3.7 PC Ameliorates Disc Degeneration in an IDD Model

**FIGURE 7:**
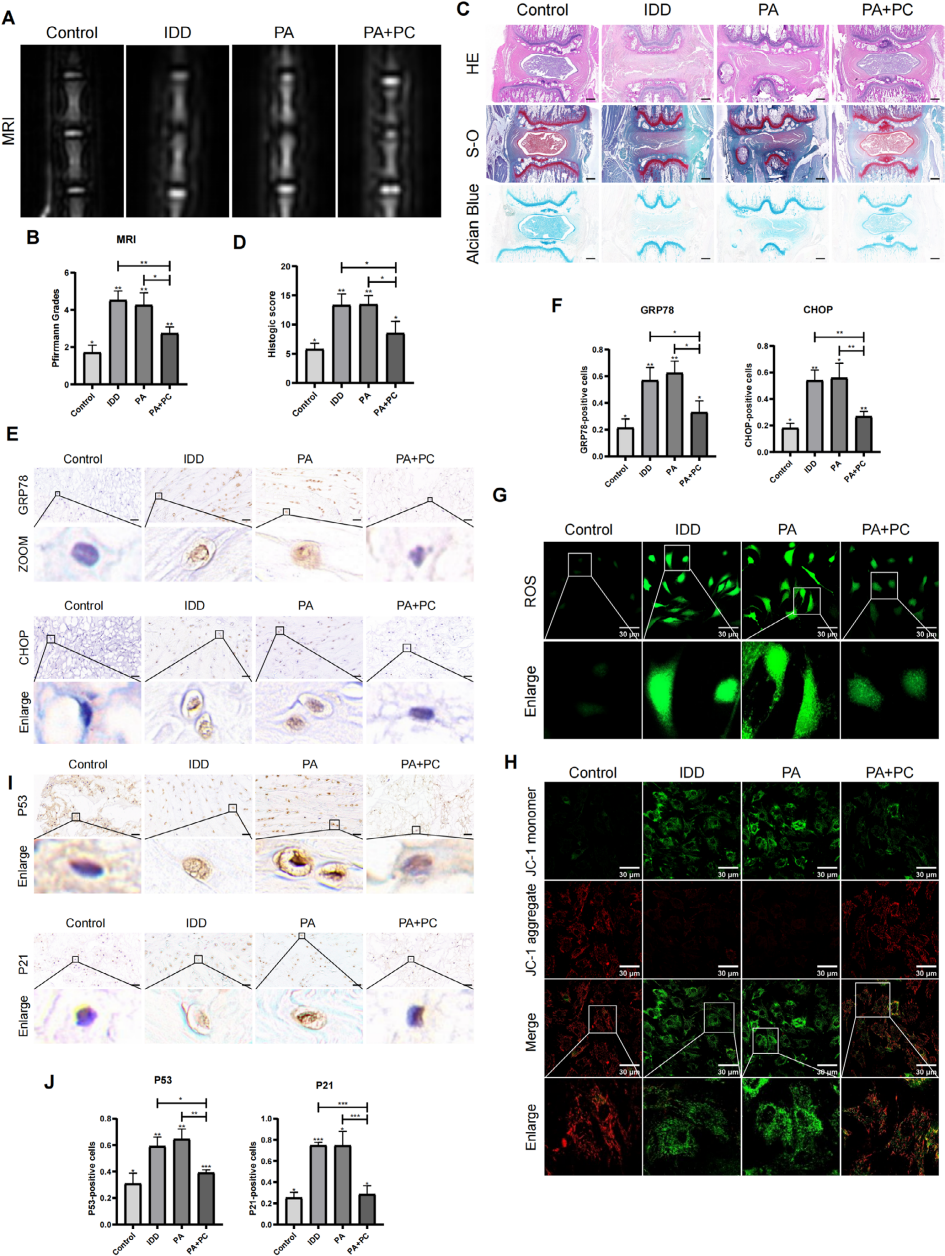
PC ameliorates disc degeneration in IDD model. (A, B) Representative T2‐weighted MRI of rat tail with a needle‐punctured and degenerated degrees were evaluated according Pfirrmann system. (C, D) The HE, S—O and Alcian blue staining of intervertebral disc postoperatively at eight weeks. The histological grades evaluated in four groups were shown in the chart. *n* = 5. ***p < 0.001. (E, F) IHC staining of GRP78 and CHOP in the intervertebral disc for each group. The rates of positive cells were calculated; *n* = 5. ***p < 0.001. (G, H) Represent images of ROS and JC‐1 staining for rat NP cells. (I, J) IHC staining of P53 and P21 in rat disc after needle‐punctured at eight weeks. The rates of positives cells were calculated. *n* = 5. **p < 0.01.

To evaluate the therapeutic effect of PC, we established an IDD model in SD rats through needle puncture or PA injection. MR images demonstrated that PA injection effectively induced IDD, in a similar manner to the commonly used needle puncture model. Furthermore, the Pfirrmann MRI grade, which reflects the extent of disc degeneration, was lower in the PC treatment group than in both the IDD and PA groups (Figure 7A,B). The histological features were analyzed via HE, S—O, and Alcian blue staining. The intervertebral disc spaces in the IDD and PA groups were distinctly narrowed, and the structure and morphology of the intervertebral disc cells in the PC group were significantly improved (Figure 7C,D). In addition, IHC staining revealed that the levels of GRP78 and CHOP were lower in the PC group than in the PA and IDD groups, confirming our in vitro results (Figure 7E,F). ROS and JC‐1 staining revealed that PC alleviated mitochondrial damage in rat NP cells (Figure 7G,H). IHC staining of P53 and P21 levels also provided evidence that PC alleviated NP cell senescence during IDD progression (Figure 7I,J). These results collectively demonstrate the effects of PC in downregulating lipid droplet accumulation and NP cell senescence, suggesting that inhibiting PA‐induced lipid droplet accumulation may represent a promising therapeutic strategy for IDD.

## 4 Discussion

Metabolism, a fundamental biological process, serves as a direct indicator of cellular, tissue, and organ function in real time [13, 23]. However, there is a limited understanding of the comprehensive metabolic profile changes that accompany NP cell senescence. As such, metabolomics can provide a detailed characterization of metabolic phenotypes and corresponding alterations in metabolism related to IDD [24]. MRS is an important functional imaging technology capable of quantifying localized chemical and metabolic shifts, making it a widely utilized tool for in vivo metabolite detection [23, 24, 25]. MRS is a noninvasive, in vivo technique for detecting endogenous tissue metabolites. In clinical settings, MRS has been extensively employed to characterize metabolic profiles within the NP and various other tissue types. The present study utilized MRS‐based metabolomics to track dynamic alterations in metabolic profiles among patients with IDD. The MRS results revealed that as the grade of disc degeneration increased, the most typical change was a greater lipid peak at 1.3 ppm, indicating abnormal lipid accumulation and lipotoxicity in IDD cases. However, these findings are inconsistent with those of prior studies indicating increased levels of certain disc chemicals, such as lactate and proteoglycans, which serve as spectroscopically quantifiable biomarkers for discogenic pain and IDD [23]. Understanding the etiology of lipotoxicity in NP cells and elucidating its underlying pathological mechanisms have become crucial areas of scientific inquiry for the effective management of IDD.

Lipotoxicity is a condition of cellular stress characterized by the accumulation of detrimental lipids within cells, resulting in dysfunction of organelles, cellular injury, or cell death [15, 26]. These harmful lipids consist of a diverse array of molecular species, such as triglycerides, free fatty acids, cholesterol, lysophosphatidylcholine, and ceramides [14]. To increase the comprehensibility of the findings obtained through MRS analysis, we conducted untargeted LC‐MS‐based metabolomic analysis on surgically harvested NP tissues, with a special focus on lipid components. In contrast to early‐stage IDD, advanced‐stage cases presented a greater abundance of 31 metabolites and a lower abundance of 23 metabolites. Notably, significantly elevated levels of PA and TG were detected in samples from advanced‐stage IDD patients. Exposure to PA has been shown to induce endoplasmic reticulum stress, mitochondrial injury, lipid accumulation, lipotoxicity, and senescence in NP cells [4]. The direct toxic effects of these harmful lipids, as well as those resulting from lipotoxicity, may both play a role in the pathogenesis of IDD. Through the integration of MRS and untargeted LC/MS‐based metabolomics analysis, we identified a close association between lipotoxicity and IDD.

The intricate and interconnected nature of lipid metabolism processes poses challenges in the use of a single omics dataset for a comprehensive analysis of the complex mechanisms underlying lipotoxicity. A combined analysis of transcription and metabolism enables a dual approach to investigate biological events, simultaneously examining both causal factors and resulting effects while identifying key genes, metabolites, and metabolic pathways involved in IDD at various levels of regulation [16, 27]. In the present investigation, complementary transcriptomic analyses were conducted, revealing significant enrichment of pathways related to lipid metabolism and cell senescence among the DEGs. Specifically, the KEGG analysis revealed that the “P53 signalling pathway,” “fatty acid synthesis,” “fatty acid degradation,” and “glycerophospholipid metabolism” pathways were significantly enriched. Integration of the transcriptomic and metabolomic data indicated that both the differentially expressed genes and the differentially abundant metabolites were collectively involved in the “glycerophospholipid metabolism” pathway, with a decrease in the abundance of the metabolites of PC.

The glycerophospholipid metabolism pathway is involved mainly in the biochemical processes involved in the synthesis, breakdown, and regulation of glycerophospholipids [28, 29]. Glycerophospholipids consist of various fatty acids and a glycerol backbone that are essential components of the biomembrane system. The common biomembrane lipid components include PC, phosphatidylethanolamine, phosphatidylserine, and sphingomyelin [30]. These complex molecules play crucial roles in maintaining the structural integrity and functionality of intracellular organelles, as well as the whole biomembrane system. Through an integrative transcriptomic and metabolomic approach, we conducted a comprehensive analysis to investigate the changes in lipid composition in advanced‐stage NP cells. Our findings revealed that PC was significantly downregulated in these cells compared with early‐stage IDD cells. PCs are essential structural lipids found in eukaryotic membranes that play crucial roles in maintaining membrane integrity and fluidity [31]. The downregulation of PC observed in advanced‐stage NP cells suggests potential alterations in cellular processes associated with membrane function and lipid metabolism. This finding may have implications for understanding the pathogenesis of lipotoxicity or stress conditions related to IDD.

PA plays a crucial role in the development and progression of IDD. In the present study, we observed aberrant accumulation of PA in NP cells from patients with advanced‐stage IDD, which subsequently led to endoplasmic reticulum stress and mitochondrial injury, as well as lipid droplet accumulation and cellular senescence [4]. This finding is consistent with previous studies that confirmed that the detrimental effects of PA manifest primarily through intracellular organelle‐mediated pathways. Xu et al. [32] provided evidence that PA induced activation of the mitochondrial apoptosis pathway through Bcl‐2 inhibition, promoted Bax, and subsequently caused Caspase3 activation in mouse spermatogonial stem cells. In addition, PA can induce cellular oxidative stress by impairing the normal function of the mitochondrial oxidative respiratory chain. Similarly, Cunha et al. [33] noted that exposure to PA induced endoplasmic reticulum stress in human islet beta cells, leading to the activation of downstream pathways such as the ATF6, IRE1α‐xbp1, and Perk pathways, ultimately culminating in cellular apoptosis. These findings suggest that reducing abnormal levels of PAs within cells can effectively mitigate damage to the cell biomembrane system. Furthermore, PA has been documented as significantly altering cellular membrane fluidity [34]. The incorporation of PA into membrane phospholipids can lead to a decrease in membrane fluidity, which is crucial for various cellular functions, including organelle biogenesis, formation signaling, and transport processes. This alteration in membrane properties can impact the activity of membrane‐associated proteins and enzymes, thereby influencing cellular metabolism and signaling pathways [35]. Thus, in our ongoing investigation, we hypothesize that the addition of PC may partially mitigate the lipotoxic effects of PA.

The aggregation of lipid droplets is a prominent characteristic of lipid toxicity in NP cells. Theoretically, the degradation of lipid droplets is a highly dynamic process. The central region of lipid droplets consists primarily of neutral lipids, specifically triglycerides and cholesterol esters [36]. Triglycerides within lipid droplets can be hydrolysed by a series of cytoplasmic lipases in a process called lipolysis, which releases glycerol and free fatty acids within the cytoplasm [37]. The regulation of free fatty acid levels within cells is predominantly governed by the processes of mitochondrial β‐oxidation and cellular transport [38]. In addition to adipocytes, other cell types possess a limited capacity for fatty acid storage [14]. Short‐ and medium‐chain fatty acids possess high membrane permeability, which enables them to pass freely through the cell membrane. In contrast, long‐chain fatty acids, such as PAs, require carrier proteins for transport across cell membranes due to their low water solubility [39]. In instances where extracellular transport is hindered, β‐oxidation serves as an efficient alternative mechanism to maintain the intracellular levels of free fatty acids [40]. The lipotoxicity induced by PA results in mitochondrial damage, ultimately leading to a diminished capacity for free fatty acid consumption; this creates a vicious cycle that exacerbates lipid toxicity.

In the present study, the utilization of a complementary transcriptomics and metabolomics approach revealed that the downregulation of PC is implicated in lipid toxicity during the progression of IDD. A reduction in PC content induces alterations in membrane components, resulting in impaired mitochondrial respiratory chain function and decreased oxidative capacity. As our results indicate, mitochondrial ROS levels increase, the membrane potential decreases, and a range of mitochondrial regulatory behaviors, such as “mitochondrial translation,” “mitochondrial translation initiation,” “mitochondrial translation elongation,” and “mitochondrial translation termination,” are abnormal. The impairment of mitochondrial function is thus a crucial determinant of the development of lipid toxicity induced by PA in NP cells. PC is a widely used drug in clinical practice, but the therapeutic effects of PC appear to be cell‐ or tissue type dependent. For example, in ulcerative colitis, PCs exert their functions by establishing a protective hydrophobic layer on the mucosal surface [41]. In adipose and muscle tissues, PC facilitates adipocyte‐specific lipolysis and apoptosis through the TNFα and IL‐1β pathways [42]. However, a different study revealed that PC has antiapoptotic effects through the inhibition of endoplasmic reticulum stress or the promotion of autophagy [43]. Here, we provide direct evidence, both in vivo and in vitro, that PC protects against lipotoxicity induced by PA through mitigating mitochondrial damage.

The findings of our study have demonstrated that PC enhances the number of contacts between the ER and mitochondria. The sites of physical communication between the endoplasmic reticulum and mitochondria, known as mitochondria‐associated ER membranes, play crucial roles in determining cell survival and death by facilitating the transfer of calcium ions and other metabolites [44, 45]. In addition, this subdomain of the ER is responsible for the biosynthesis of two abundant phospholipids, PC and phosphatidylethanolamine. In addition to their synthesis, there is also extensive lipid exchange between the ER and mitochondrial membranes: PC is the most abundant mitochondrial phospholipid, and together with PS, it must be imported from the ER [46]. In conjunction with their synthesis, there is a significant exchange of lipids between the membranes of the ER and mitochondria. PC, along with phosphatidylserine, is the most abundant phospholipid among the mitochondrial membrane components and must be imported from the ER. The function or dysfunction of one organelle can have a profound impact on the other; however, the significance of this interaction in relation to PA‐induced cellular dysfunction and lipid toxicity remains largely unexplored. Herein, we demonstrated that PC protects mitochondrial function against PA‐induced stress conditions, as evidenced by the reduction in ROS levels, enhancement of the MMP, and promotion of mitochondrial biogenesis and fatty acid oxidation. However, the protective effects of PC on PA‐induced mitochondrial dysfunction were nullified in the presence of tunicamycin, an ER stress inducer, suggesting that PC protects against PA‐induced lipid toxicity through direct interactions between the ER and mitochondria.

IDD is the primary etiology of chronic low back pain, representing a significant public health concern associated with substantial societal, economic, and familial burdens [1]. Given the limitations of the efficacious pharmacological treatments available, it has become imperative to focus on targeted interventions for this debilitating condition. Notably, our study integrates multiomics data with ultrastructural and functional assessments to offer mechanistic insights into the involvement of lipid toxicity in NP cell senescence and to unveil the therapeutic potential of PC in IDD management. In nonadipose cells, lipid toxicity is a stress condition characterized by the accumulation of harmful lipids, leading to dysfunction of intracellular organelles, cellular injury, or senescence. By employing a multiomics methodology, we identified a significant pathological lipid toxicity condition associated with IDD. Importantly, PC has emerged as an affordable and easily accessible pharmaceutical agent. The PC synthesis reported herein presents potential molecular targets and therapeutic agents for IDD therapy.

## Author Contributions

Conceptualization, X.C. and W.Z.; sample collection and processing, L.D. and Y.D.; analysis and integration of multi‐omics data, S.T. and X.C.; development of methodology, M.J., C.L., and X.C.; writing, review, and/or revision of the manuscript: X.C.

## Conflicts of Interest

The authors declare no conflicts of interest.

## Supporting information


**Figure S1.** Transcriptomics analysis for IDD. (A) KEGG analysis for the subset of down‐regulated genes; (B) KEGG analysis for the subset of up‐regulated genes; (C–F) GSEA analysis identified the significant items.

**Figure S2.** PA results in endoplasmic reticulum stress and mitochondrial damage. (A–D) PA increased in expression of GRP 78 and CHOP; *n* = 3. ***p < 0.001. Scale bar: 10 μm. (E) IF staining of TOMM20 for mitochondrial observation after PA treatment. Scale bar: 10 μm. (F–G) PA results in the increased level of ROS. *n* = 3. ***p < 0.001. Scale bar: 30 μm. (H–I) The JC‐1 staining and comparison of the relative mean fluorescence intensity between different groups. *n* = 3. ***p < 0.001. Scale bar: 30 μm. (J–K) Bodipy staining and statistical analysis for lipid droplets in NP cells. *n* = 3. ***p < 0.001. Scale bar: 10 μm. (L) TEM observation for the lipids droplet. Scale bar: 500 nm.

**Table S1.** Participants information for magnetic resonance spectroscopy (MRS).

## Data Availability

Data will be available on reasonable request to the corresponding author.
