# Supplementary figures and images for: Phosphatidylcholine Ameliorates Palmitic Acid‐Induced Lipotoxicity by Facilitating Endoplasmic Reticulum and Mitochondria Contacts in Intervertebral Disc Degeneration
Source: JOR Spine. 2025 Mar 31;8(2):e70062. doi: 10.1002/jsp2.70062 (PMC11956213; doi:10.1002/jsp2.70062)

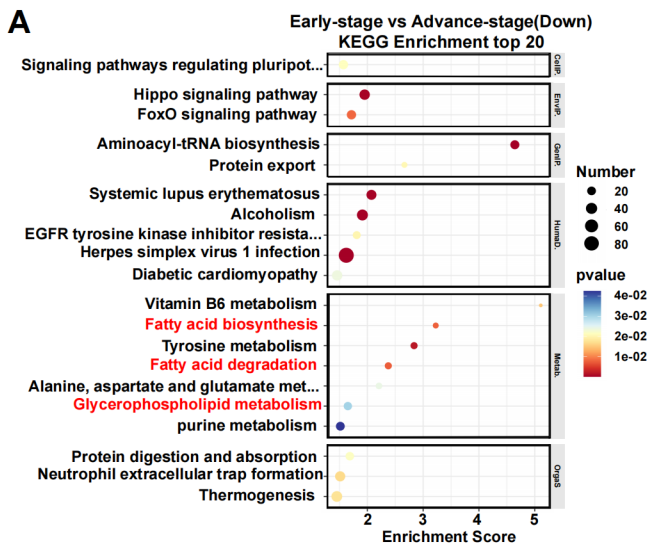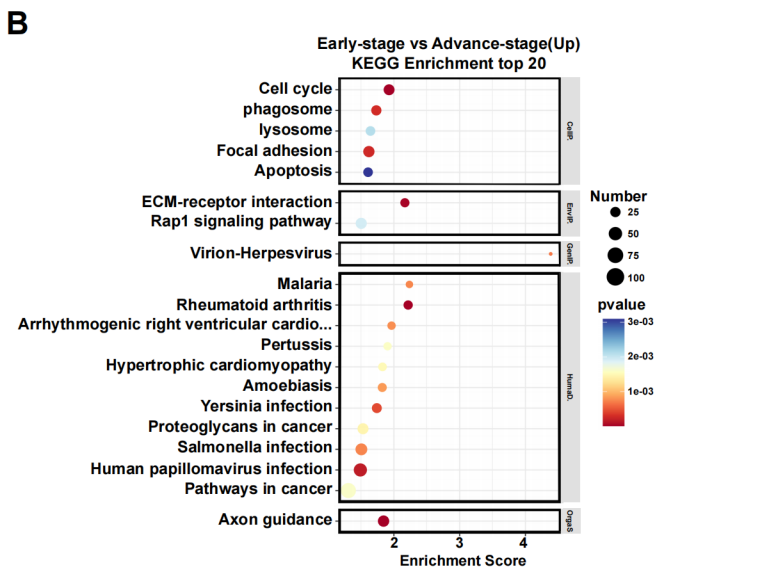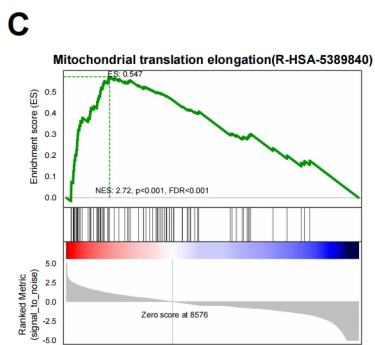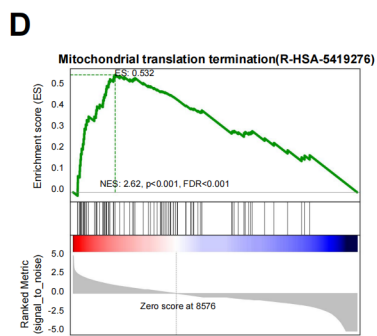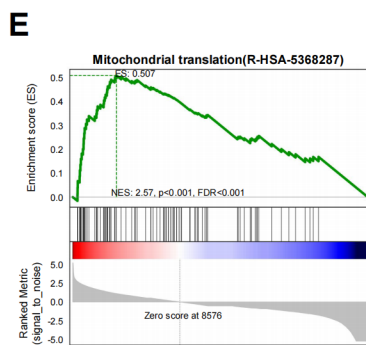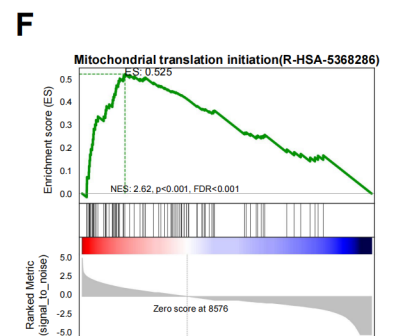

Supplement: Supplementary file 1 — Figure S1. Transcriptomics analysis for IDD. (A) KEGG analysis for the subset of down‐regulated genes; (B) KEGG analysis for the subset of up‐regulated genes; (C–F) GSEA analysis identified the significant items. [file JSP2-8-e70062-s002.pdf]

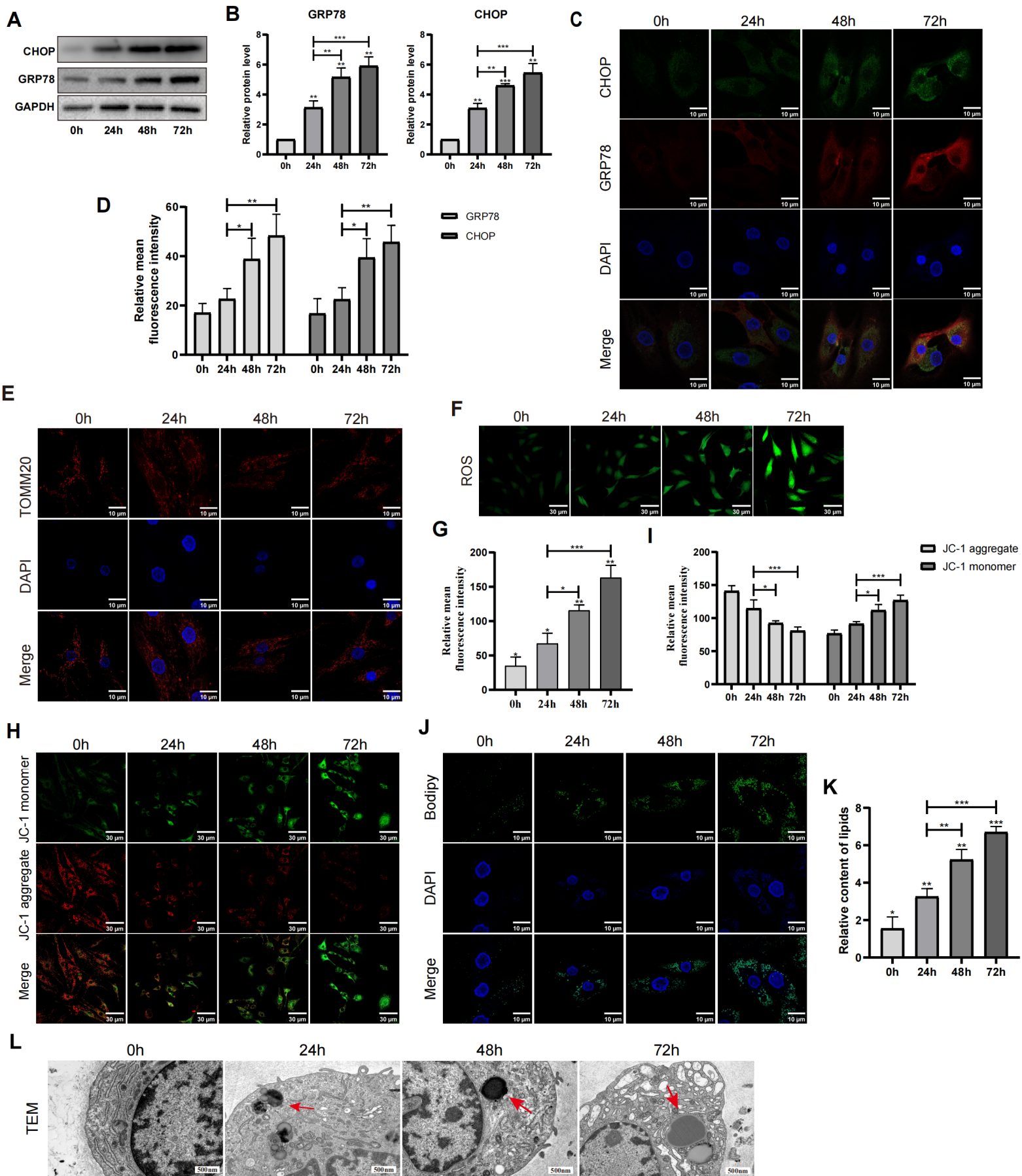

Supplement: Supplementary file 2 — Figure S2. PA results in endoplasmic reticulum stress and mitochondrial damage. (A–D) PA increased in expression of GRP 78 and CHOP; n = 3. ***p < 0.001. Scale bar: 10 μm. (E) IF staining of TOMM20 for mitochondrial observation after PA treatment. Scale bar: 10 μm. (F–G) PA results in the increased level of ROS. n = 3. ***p < 0.001. Scale bar: 30 μm. (H–I) The JC‐1 staining and comparison of the relative mean fluorescence intensity between different groups. n = 3. ***p < 0.001. Scale bar: 30 μm. (J–K) Bodipy staining and statistical analysis for lipid droplets in NP cells. n = 3. ***p < 0.001. Scale bar: 10 μm. (L) TEM observation for the lipids droplet. Scale bar: 500 nm. [file JSP2-8-e70062-s001.pdf]
