# Supplementary material for: Phosphatidylcholine Ameliorates Palmitic Acid‐Induced Lipotoxicity by Facilitating Endoplasmic Reticulum and Mitochondria Contacts in Intervertebral Disc Degeneration
Source: JOR Spine. 2025 Mar 31;8(2):e70062. doi: 10.1002/jsp2.70062 (PMC11956213; doi:10.1002/jsp2.70062)
Supplement: Supplementary file 3 — Table S1. Participants information for magnetic resonance spectroscopy (MRS). [file JSP2-8-e70062-s003.pdf]

**Supplementary table 1. Participants information for MRS**

| NO. | **Sex** | **Age (year)** | **Pfirrmann Grade** | **Group** |
|---|---|---|---|---|
| 1 | Male | 35 | I | Healthy volunteers |
| 2 | Male | 25 | I | Healthy volunteers |
| 3 | Female | 29 | I | Healthy volunteers |
| 4 | Male | 27 | I | Healthy volunteers |
| 5 | Male | 22 | I | Healthy volunteers |
| 6 | Female | 24 | I | Healthy volunteers |
| 7 | Male | 34 | IV | IDD |
| 8 | Male | 55 | V | IDD |
| 9 | Male | 40 | IV | IDD |
| 10 | Female | 56 | III | IDD |
| 11 | Female | 55 | IV | IDD |
| 12 | Female | 56 | V | IDD |

**Supplementary Figure 1** Transcriptomics analysis for IDD. (A) KEGG analysis for the subset of down-regulated genes; (B) KEGG analysis for the subset of up-regulated genes; (C~F) GSEA analysis identified the significant items.

**Supplementary Figure 2** PA results in endoplasmic reticulum stress and mitochondrial damage. (A~D) PA increased in expression of GRP 78 and CHOP; n=3. ***P<0.001. Scale bar: 10μm. (E) IF staining of TOMM20 for mitochondrial observation after PA treatment. Scale bar: 10μm. (F~G) PA results in the increased level of ROS. n=3. ***P<0.001. Scale bar: 30μm. (H~I) The JC-1 staining and comparison of the relative mean fluorescence intensity between different groups. n=3. ***P<0.001. Scale bar: 30μm. (J~K) Bodipy staining and statistical analysis for lipid droplets in NP cells. n=3. ***P<0.001. Scale bar: 10μm. (L) TEM observation for the lipids droplet. Scale bar: 500 nm.
